# Structural and functional characterization of the IpaD π-helix reveals critical roles in DOC interaction, T3SS apparatus maturation, and *Shigella* virulence

**DOI:** 10.1016/j.jbc.2024.107613

**Published:** 2024-07-28

**Authors:** Samuel A. Barker, Abram R. Bernard, Yalemi Morales, Sean J. Johnson, Nicholas E. Dickenson

**Affiliations:** Department of Chemistry and Biochemistry, Utah State University, Logan, Utah, USA

**Keywords:** bacterial pathogenesis, bile acid, cell invasion, flow cytometry, fluorescence anisotropy, host-pathogen interaction, infectious disease, ligand-binding protein, secretion, type III secretion system (T3SS), X-ray crystallography

## Abstract

*Shigella* spp. are highly pathogenic members of the Enterobacteriaceae family, causing ∼269 million cases of bacillary dysentery and >200,000 deaths each year. Like many Gram-negative pathogens, *Shigella* rely on their type three secretion system (T3SS) to inject effector proteins into eukaryotic host cells, driving both cellular invasion and evasion of host immune responses. Exposure to the bile salt deoxycholate (DOC) significantly enhances *Shigella* virulence and is proposed to serve as a critical environmental signal present in the small intestine that prepares *Shigella’s* T3SS for efficient infection of the colonic epithelium. Here, we uncover critical mechanistic details of the *Shigella*-specific DOC signaling process by describing the role of a π-helix secondary structure element within the T3SS tip protein invasion plasmid antigen D (IpaD). Biophysical characterization and high-resolution structures of IpaD mutants lacking the π-helix show that it is not required for global protein structure, but that it defines the native DOC binding site and prevents off target interactions. Additionally, *Shigella* strains expressing the π-helix deletion mutants illustrate the pathogenic importance of its role in guiding DOC interaction as flow cytometry and gentamycin protection assays show that the IpaD π-helix is essential for DOC-mediated apparatus maturation and enhanced invasion of eukaryotic cells. Together, these findings add to our understanding of the complex *Shigella* pathogenesis pathway and its evolution to respond to environmental bile salts by identifying the π-helix in IpaD as a critical structural element required for translating DOC exposure to virulence enhancement.

*Shigella* spp. are nonmotile Gram-negative bacteria that cause a severe form of bacillary dysentery (shigellosis) in humans. As enteric pathogens, *Shigella* are generally transmitted *via* the fecal oral route where they efficiently pass through the stomach and small intestine to invade the colonic epithelium. From here, the infection results in cytokine-mediated inflammation of the colon and necrosis of the colonic epithelium which produces the hallmark symptoms of shigellosis that can be fatal in children, the elderly, and the immunocompromised (1, 2). Furthermore, *Shigella* are exceptionally virulent, requiring exposure to as few as 10 to 100 bacteria for the onset of infection in a healthy adult (3), leading to an estimated 269 million annual worldwide infections and >200,000 deaths each year (4). This threat is further exacerbated by the lack of a Food and Drug Administration approved vaccine against *Shigella* and the rapid emergence of multidrug-resistant and extensively drug resistant strains that can no longer be treated by many of the antibiotics traditionally prescribed (https://www.who.int/emergencies/disease-outbreak-news/item/2022-DON364) (https://emergency.cdc.gov/han/2023/han00486.asp) (5). Together, these factors have forced a “call to arms” from both the US Centers for Disease Control and World Health Organization for research that describes the mechanistic details of *Shigella* pathogenesis and provides new means for preventing/combatting infection (https://emergency.cdc.gov/han/2023/han00486.asp) (https://www.who.int/teams/immunization-vaccines-and-biologicals/diseases/shigella) (https://www.cdc.gov/drugresistance/biggest-threats.html).

*Shigella* virulence is absolutely reliant upon a single type three secretion system (T3SS) that supports both invasion of the hosts’ colonic epithelium and evasion of resulting immune responses (6, 7, 8). Its T3SS is composed of >30 different proteins, many of which make up the syringe and needle-like apparatus (injectisome) that facilitates injection of effector proteins from the bacterial cytoplasm into host cells (6, 7). The injectisome can be described as containing four distinct regions: the cytoplasmic sorting platform which is believed to be responsible for recognition and partial unfolding of proteins in preparation for secretion, the basal body which spans the inner and outer bacterial membranes and anchors the apparatus to the bacteria, the hollow extracellular needle which extends from the basal body past the lipopolysaccharide layer, and the needle-tip complex which terminates the injectisome structure and forms the translocon pore within the host cell membrane (7, 9, 10). Once this conduit is complete, an arsenal of ∼25 to 30 different effector proteins is secreted into the host cell, supporting infection and subverting host immune responses (11, 12, 13).

*Shigella* is not the only bacteria to express a T3SS as many other plant, animal, and human pathogens also rely on one or more T3SSs as primary virulence factors (8, 14, 15) and similarities among injectisome structures frequently lead to better understanding of the system(s) in related bacteria. It is important to recognize, however, that each T3SS has evolved to best support the infection niche of the pathogen expressing them. For example, secreted *Shigella* effectors support critical roles including actin polymerization-induced membrane ruffling leading to bacterial uptake (16, 17) as well as rupture of the resulting vacuole to allow replication within the host cell cytoplasm and cell-to-cell spread (9, 16). *Salmonella*, however, express two T3SSs with *Salmonella* pathogenicity island-1 also supporting invasion *via* actin polymerization and *Salmonella* pathogenicity island--2 subsequently supporting the formation and maintenance of the *Salmonella*-containing vacuole in which replication primarily occurs (18, 19). In addition to differences in effector roles, numerous studies have identified specific environmental signals that influence pathogen virulence through impact on T3SS transcription (20, 21, 22, 23, 24), translation (25), tip complex maturation (7, 26, 27, 28), and regulation of protein secretion through the injectisome (29, 30, 31). These environmental signals and the responses to them are again specific to the infection niche of the bacteria that express them.

Studies in *Shigella* have demonstrated that bile salts found in the small intestine (*e.g.*, deoxycholate (DOC)) interact directly with the injectisome tip complex protein invasion plasmid antigen D (IpaD) to support interaction with the hydrophobic translocator protein IpaB and its stable recruitment to the maturing tip complex (26, 32, 33, 34). Now primed with both IpaD and IpaB, the injectisome is poised for interaction with the host cell membrane where exposure to cholesterol and sphingomyelin in the host cell membrane efficiently recruit the translocator protein IpaC to the tip complex (29). Recruitment of IpaC represents the final maturation step, completing the translocon pore in the host membrane, initiating protein effector secretion into the host cell cytoplasm, and inducing macropinocytosis as the first step of cellular invasion by *Shigella* (7, 29). This work identified what continues to be the only complete stepwise maturation process in any T3SS, perhaps driving the extraordinary efficiency with which *Shigella* infects its hosts.

More recently, while describing the mechanistic influence of DOC on *Shigella* virulence enhancement, we identified a π-helix secondary structure element in IpaD that is integral in apparatus maturation and appears to hold the key to understanding how DOC influences *Shigella* virulence (34, 35). π-helices are structurally related to α-helices, but result from a local i + 5 hydrogen bonding pattern as opposed to i + 4 seen in an α-helix (36, 37). The resulting local structure of the π-helix is thought to evolutionarily derive from the insertion of an amino acid into an α-helix, providing a higher free energy structure that tends to provide additional structural flexibility and is often associated with critical protein function(s) (36, 38). Here, we directly assess the role of the π-helix in IpaD by engineering mutants that convert it to an α-helix without otherwise significantly affecting the protein structure. High-resolution crystal structures of wild-type IpaD (IpaD^WT^) and one of the engineered IpaD π-helix deletion mutants in both apo and DOC bound conditions uncovers clear structural implications for the role of the π-helix in regulating and communicating DOC binding. Leveraging *Shigella* strains expressing the IpaD π-helix deletion mutants correlates its role to injectisome tip maturation and cellular invasion phenotype with the findings presented here providing a critical description of the molecular basis for the IpaD π-helix and DOC influence on *Shigella* virulence that appears to have uniquely evolved to support DOC-mediated virulence enhancement in *Shigella*.

## Results

### Conversion of the IpaD π-helix into an α-helix does not impact DOC binding *in vitro*

We have previously described the native π-helix structure in IpaD and shown that some mutations in this region negatively impact DOC-mediated virulence enhancement in *Shigella*, suggesting that the π-helix plays a vital role in this critical virulence enhancement mechanism (34, 35). Further probing the role of the π-helix in DOC interaction/virulence enhancement, however, required that we engineer an IpaD mutant(s) lacking the π-helix structure altogether. Careful consideration of the WT structure suggested that the localized i + 5 hydrogen bonding pattern of the π-helix results from the “insertion” of an amino acid into this region and that removing either the glutamine at position 148 or the tyrosine at position 149 would eliminate the π-helix and return it to a continuous i + 4 hydrogen bonding α-helix. Fortunately, both the IpaD^ΔQ148^ and IpaD^ΔY149^ constructs expressed well in *Escherichia coli* and purified nearly identically to IpaD^WT^.

**Figure 1:**
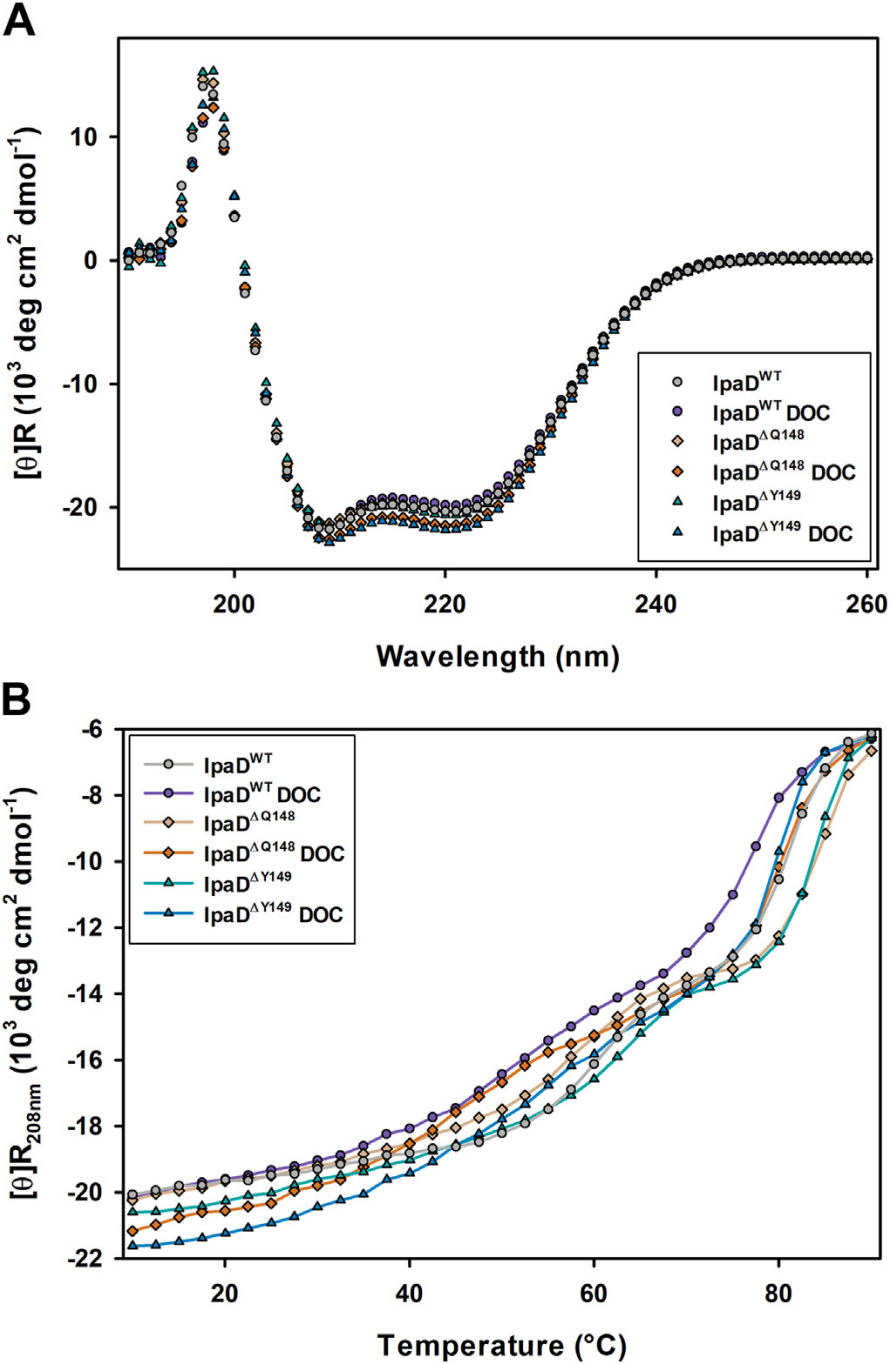
**CD analysis of IpaD**^**WT**^**and the engineered IpaD**^**ΔQ148**^**and IpaD**^**ΔY149**^**π-helix deletion mutants.***A*, far-UV CD spectra of each construct in the absence and presence of DOC demonstrate nearly identical CD spectra intensities with local minima at 208 nm and 222 nm, consistent with the predominantly α-helical secondary structures observed for these constructs. *B*, thermal unfolding profiles result from plotting the mean residue molar ellipticity as a function of solution temperature. The two thermal transitions observed for each construct are expected and consistent with the unfolding of the protein distal domain followed by loss of the global secondary structure. The presence of DOC decreased the Tm of each construct while the π-helix deletion mutants both exhibited increased thermal stability compared to the IpaD^WT^ construct containing the π-helix. The included spectra and thermal profiles are representative of three independent biological replicates and the quantified results from the full data set are summarized in Table 1. DOC, deoxycholate; Ipa, invasion plasmid antigen.

CD spectroscopy provided an efficient means of preliminary structural characterization of the two IpaD mutants and a way to ensure that the mutations did not compromise the overall protein structure. The far-UV CD spectra of IpaD^WT^ and the two engineered mutants were collected in the absence and presence of DOC and are all nearly superimposable (Fig. 1*A*). Dichroweb analysis of the spectra confirms that each IpaD construct maintains the expected distribution of secondary structure content and that the deletion mutations do not significantly alter the overall secondary structure of IpaD (Table 1). CD thermal unfolding profiles were collected for each of the conditions described above, providing sensitive means of probing the thermodynamic impact of the mutations. The profile for each condition exhibits the characteristic two step unfolding mechanism attributed to the lower temperature denaturation of the distal domain and the higher temperature unfolding of the core that contains the π-helix (35, 39). Focusing on the disruption of the protein core, deletion of Q148 and Y149 each increased the thermal stability of this event from 81 ± 1 °C for IpaD^WT^ to 85 ± 1 °C and 84 ± 2 °C, respectively (Fig. 1*B* and Table 1). Interestingly, the addition of DOC modestly decreased the thermal transition temperature for each of the constructs. While it is not clear at this time whether this is a direct impact of specific DOC interaction reducing the global stability of IpaD or perhaps a less specific detergent effect by DOC, both deletion mutants maintain a higher transition temperature when compared to IpaD^WT^, consistent with the elimination of the thermodynamically less stable π-helix compared to its α-helix counterpart.

**Table 1:** CD secondary structure spectral analysis and calculated melting temperatures for WT and and π-helix deletion mutants of IpaD in the presence and absence of deoxycholate

Percent secondary structure^a^ (% ± SD)								
IpaD Construct	−DOC			+DOC			Tm^b^ (°C ± SD)	
	α-Helix	β-Sheet	Random Coil	α-Helix	β-Sheet	Random Coil	−DOC	+DOC
IpaD^WT^	63 ± 1	16 ± 1	20 ± 1	63 ± 1	16 ± 3	21 ± 3	81 ± 1	77 ± 1^c^
IpaD^ΔQ148^	64 ± 1	15 ± 5	21 ± 3	64 ± 1	14 ± 1	24 ± 1	85 ± 1^d^	82 ± 2^d^
IpaD^ΔY149^	65 ± 2	12 ± 1	22 ± 1	64 ± 1	16 ± 1	20 ± 1	84 ± 2^d^	81 ± 1^d^

a The Dichroweb spectral analysis package K2D was used to calculate secondary structure content from far-UV circular dichroism spectra of the tested IpaD constructs in the presence and absence of DOC.

b Secondary structure thermal stability was examined by measuring CD signal at 222 nm while the protein solution temperature was increased from 10 to 90 °C. All values are reported as the mean ± the standard deviation from three independent biological replicates.

c Indicates a statistical difference in Tm of the tested construct resulting from the addition of DOC.

d Indicates a statistical difference in Tm from the IpaD^WT^ construct in the respective apo or +DOC condition (one-way ANOVA followed by a Dunnett’s *post hoc* test, *p* ≤ 0.05).

**Figure 2:**
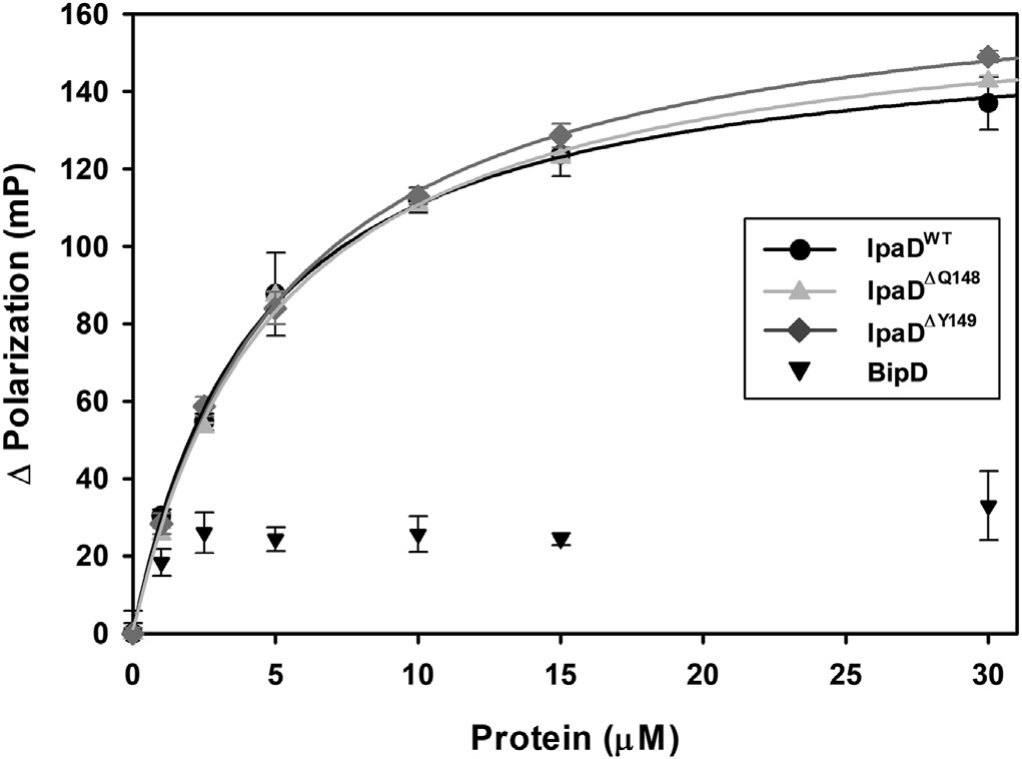
**Impact of the IpaD π-helix on deoxycholate affinity.** Purified recombinant IpaD^WT^ and the π-helix deletion constructs developed in this study (IpaD^ΔQ148^ and IpaD^ΔY149^) were titrated into FITC-DOC and polarization binding curves were generated by plotting the mean ± standard deviation of the change in polarization as a function of IpaD concentration. The *Burkholderia* T3SS tip protein, BipD, serves as a control. The results were fit to a single site saturation model, and fits resulting in a R^2^ ≥ 0.95 were overlaid on the binding data and used to calculate the apparent K_d_ of the interactions. Each plot results from triplicate technical replicates and is itself representative of three independent biological replicates. DOC, deoxycholate; Ipa, invasion plasmid antigen.

Fluorescence polarization assays provided the first quantitative description of DOC interaction with IpaD (27) and continue to provide an efficient and powerful tool for assessing the impact of the π-helix deletion mutations on DOC interaction. The fluorescence polarization binding curves presented in Figure 2 were fit to a single site saturation binding model to calculate the apparent K_d_s between DOC and the IpaD constructs. The apparent K_d_ between DOC and IpaD^WT^ was found to be 4.7 ± 1.0 μM and is consistent with previously calculated affinities reported for IpaD^WT^ (27, 35). The apparent K_d_s between DOC and IpaD^ΔQ148^ and IpaD^ΔY149^ were essentially unaltered from WT at 4.9 ± 1.0 μM and 5.2 ± 1.0 μM, respectively (Table 2). The IpaD homolog, BipD, from the *Burkholderia* T3SS serves as a control as it has previously been shown to not bind DOC efficiently (27).

**Table 2:** Fluorescence polarization-derived binding affinities between engineered IpaD π-helix mutants and deoxycholate

IpaD construct	K_d_ (μM ± SD)^a^
IpaD^WT^	4.7 ± 1.0
IpaD^ΔQ148^	4.9 ± 1.0
IpaD^ΔY149^	5.2 ± 1.0

a Apparent K_d_ values between FITC-DOC and the WT and engineered IpaD pi-helix deletion mutants used in this study. Values are reported as the mean ± standard deviation of the apparent K_d_s calculated from three independent biological replicates. No statistical difference in DOC affinity is observed (one-way ANOVA, *p* ≤ 0.05).

### Crystal structures confirm conversion of the π-helix

**Figure 3:**
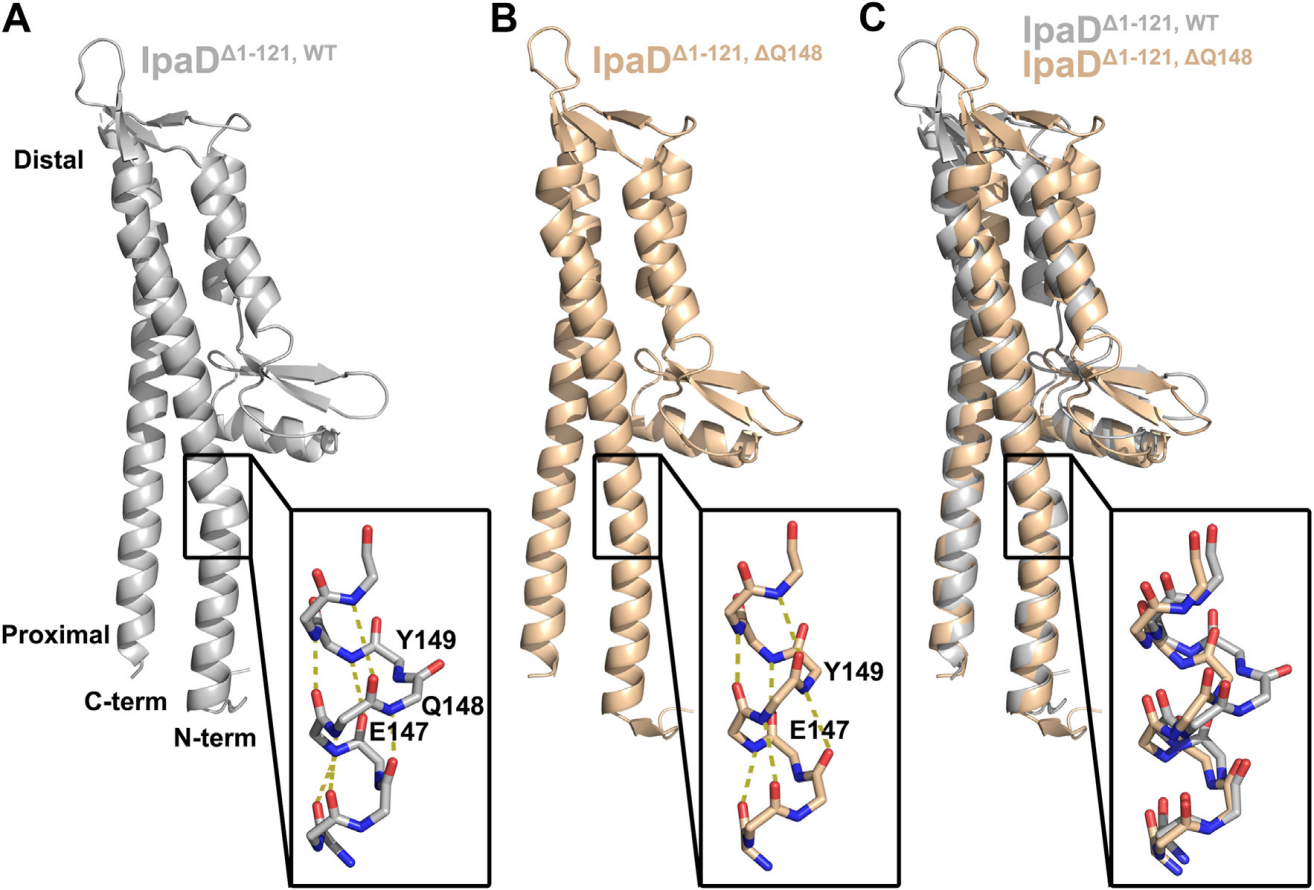
**X-ray crystal structures of IpaD**^**Δ1–121,**^^**WT**^**and the engineered π-helix deletion mutant IpaD**^**Δ1–121, ΔQ148**^**.***A*, the *gray* IpaD^Δ1–121,^^WT^ structure (8V7S) contains a short span of π-helix secondary structure visible within the overlaid *black box*. A zoomed in view of the corresponding protein backbone structure in which carbon, oxygen, and nitrogen are colored *gray*, *red*, and *blue*, respectively, identifies the i + 5 backbone hydrogen bonding pattern surrounding the helix “aneurism” containing the residues Q148 and Y149 that defines the π-helix structure. *B*, in contrast, the *wheat* colored IpaD^Δ1–121,^^ΔQ148^ structure (8V7Q) engineered to eliminate the π-helix lacks the native aneurism and displays a continuous i + 4 hydrogen bonding pattern throughout the length of the helix. *C*, a global Cα alignment helps visualize the loss of the native π-helix in the mutant structure while confirming the mutation has little impact on the overall protein structure. Ipa, invasion plasmid antigen.

A small number of groups, including our own, have crystallized IpaD and IpaD mutants and while some full-length IpaD structures have been solved (40, 41), proteolytic cleavage of the N-terminal domain facilitates crystallization (34, 40). Here, we mimicked the previously successful native proteolytic cleavage by engineering IpaD^WT^, IpaD^ΔQ148^, and IpaD^ΔY149^ to lack their N terminal 121 residues. These N-terminal truncation constructs expressed and purified well in *E. coli* and diffracting crystals of IpaD^Δ1–121, WT^ and IpaD^Δ1–121, ΔQ148^ formed overnight by vapor diffusion. Unfortunately, diffracting crystals of IpaD^Δ1–121, ΔY149^ were not achieved. Diffraction data were collected to generate crystal structures at 2.70 Å and 3.00 Å resolution for IpaD^Δ1–121, WT^ (Protein Data Bank (PDB) ID 8V7S) and IpaD^Δ1–121, ΔQ148^ (PDB ID 8V7Q), respectively (Fig. 3 and Table 3). The IpaD^Δ1–121, WT^ *structure* aligns well with the previously published proteolytically truncated IpaD structure (PDB ID 8V7Q) (40) with a Cα RMSD of 0.485 Å (Fig. S1). The IpaD^Δ1–121, ΔQ148^ structure clearly shows that the π-helix has been eliminated through conversion of the surrounding region to an α-helix. When the proximal (bottom) regions of the structures are aligned, a modest translation of the distal portion of the structure is observed in the mutant relative to that of the WT. Importantly, however, elimination of the π-helix does not impact the registry of the surrounding residues or significantly alter the positioning of the adjacent side chains (Fig. 3*C*), supporting the feasibility of this mutant as a means to independently dissect the role of the π-helix in IpaD. Unfortunately, we were unable to structurally confirm the impact of deleting Y149 from IpaD. However, the similarity in relationship of the Q148 and Y149 residues within the i + 5 hydrogen bonding region of the π-helix suggest that the IpaD^ΔY149^ construct likely shares structural characteristics, such as removal of the π-helix, with the solved IpaD^Δ1–121, ΔQ148^ structure and both mutations are therefore considered throughout this study.

**Table 3:** Data collection, phasing, and refinement statistics

	IpaD^Δ1–121, WT^	IpaD^Δ1–121, WT^ DOC-bound	IpaD^Δ1–121, ΔQ148^	IpaD^Δ1–121, ΔQ148^ DOC-bound
Data collection				
Beamline	SSRL 9-2	SSRL 9-2	SSRL 9-2	SSRL 9-2
Wavelength	0.9795	0.9795	0.9795	0.9795
Space group	P2_1_	P2_1_	P2_1_	P 2_1_ 2_1_ 2_1_
Cell dimensions				
a, b, c (Å)	62.6, 44.0, 92.5	62.8, 43.4, 93.4	45.0, 69.4, 173.0	43.6, 70.3, 171.2
α, β, γ (°)	90.0, 97.2, 90.0	90.0, 97.4, 90.0	90.0, 90.3, 90.0	90.0, 90.0, 90.0
Resolution (Å)^a^	50.00–2.70 (2.80–2.70)	34.06–1.96 (2.03–1.96)	50.00–3.00 (3.11–3.00)	34.44–2.20 (2.28–2.20)
No. of reflections	13,325	35,231	20,795	26,865
CC½ (outer shell)	0.824	0.948	0.602	0.846
I/σ	11.88 (1.62)	17.97 (4.75)	19.57 (1.13)	30.84 (2.73)
Completeness (%)	95.56 (80.55)	97.21 (91.02)	95.76 (89.88)	97.35 (90.84)
Redundancy	5.0 (3.7)	6.6 (5.6)	6.2 (4.1)	12 (6.7)
Refinement				
Resolution (Å)	45.89–2.70 (2.8–2.7)	34.06–1.96 (2.03–1.96)	28.83–3.00 (3.11–3.00)	34.44–2.20 (2.28–2.20)
R_work_/R_free_	0.225/0.286	0.189/0.224	0.257/0.271	0.197/0.230
No. of molecules	2	2	4	2
No. of atoms				
Protein	3008	2950	6000	3030
Ligand/ion	-	68	-	62
Water	23	146	-	91
B-factors				
Protein	64.61	63.09	89.89	64.22
Ligand/ion	-	63.92	-	81.56
RMSD				
Bond lengths (Å)	0.002	0.009	0.002	0.002
Bond angles (°)	0.51	0.88	0.41	0.42
Ramachandran				
Preferred (%)	96.8	97.6	97	97.4
Outliers (%)	0.26	0	0.13	0
PDB code	8V7S	8V5C	8V7Q	8V5E

a Values in parenthesis are for the highest resolution shell.

### The IpaD π-helix is essential for DOC-enhancement of *Shigella* virulence, but not for IpaD’s roles in regulating uninduced or Congo red-induced protein secretion through the T3SS apparatus

**Figure 4:**
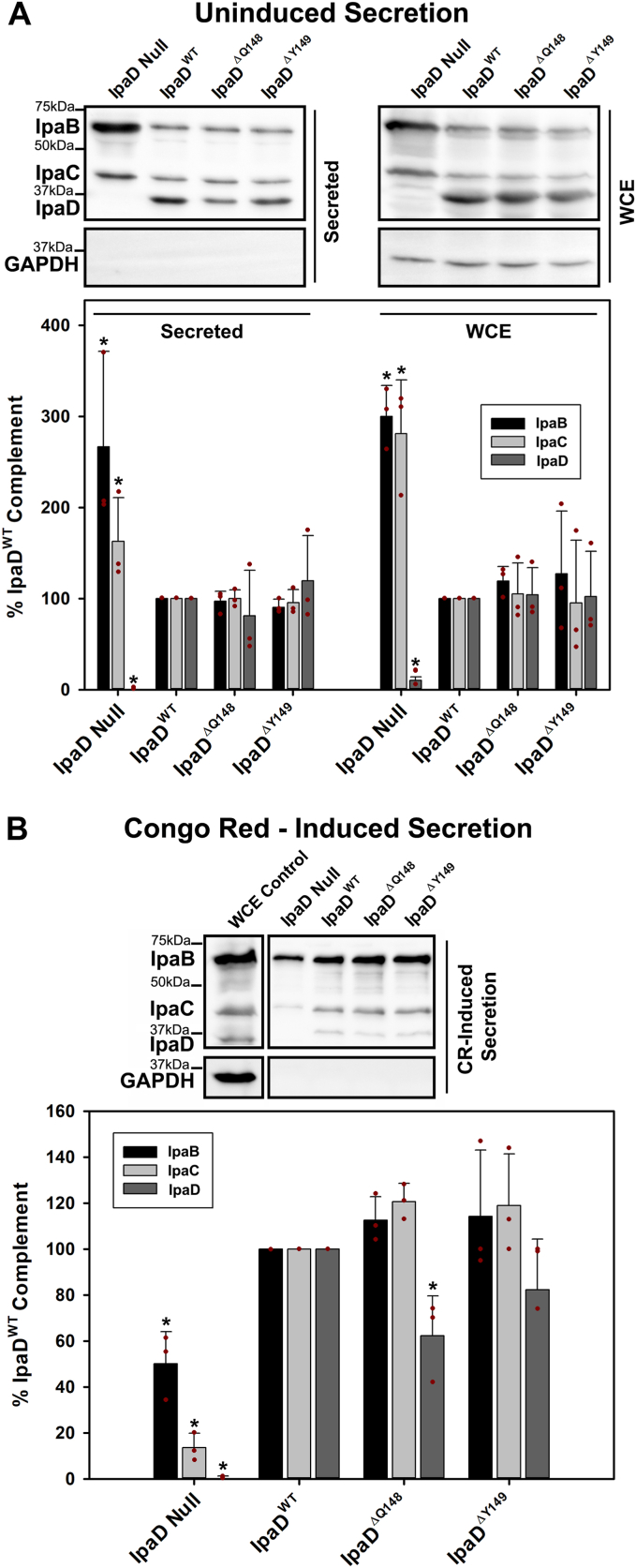
**IpaD π-helix deletion mutants retain their function in controlling T3SS protein secretion.** Representative Western blots and resulting densitometry comparisons probing the impact of the engineered IpaD^ΔQ148^ and IpaD^ΔY149^ mutations on (*A*) uninduced and (*B*) Congo red induced secretion of T3SS proteins. In the uninduced profiles, both the overnight culture supernatant (*left*) and *Shigella* whole-cell extracts (WCE, *right*) were examined. In the Congo red induced profiles, the culture supernatants were probed following T3SS activation by Congo red. In both experiments, the cytoplasmic control protein GAPDH confirms that the Ipa proteins observed in the supernatant are the result of protein secretion and are not the result of cell lysis and also serves as a loading control in the WCE blot. The mean ± standard deviation of the Western blot band intensities from three independent biological replicates are plotted relative to the IpaD^WT^ control strain. ∗ denotes a statistical difference compared to the detected level of the same protein from the *Shigella* strain expressing IpaD^WT^ [one-way ANOVA followed by a Dunnett’s post test (*p* ≤ 0.05)]. Ipa, invasion plasmid antigen; T3SS, type three secretion system.

After confirming the overall integrity of the IpaD protein mutants and verifying the elimination of the π-helix in the IpaD^ΔQ148^ construct through X-ray crystallography, we engineered *Shigella* strains expressing the IpaD π-helix mutants to directly assess the role of IpaD’s π-helix in *Shigella* virulence phenotype. In *Shigella*, IpaD resides at the extracellular tip of the injectisome where it functions as a “gatekeeper” that prevents unwanted protein secretion (leakage) through the injectisome prior to requisite activation events. As described previously, strains lacking IpaD fail to control this unwanted secretion, resulting in loss of cytoplasmic T3SS effector and translocator proteins to the growth media (28, 33, 42, 43). An uninduced protein secretion assay determines IpaD’s ability to function in this regulatory capacity by growing *Shigella* cultures in liquid media overnight, precipitating secreted proteins from the media, and detecting several select T3SS proteins *via* Western blot (42, 44, 45). Figure 4*A* shows that *Shigella* strains expressing the π-helix deletion mutants share similar levels of protein expression to a strain expressing IpaD^WT^ and that there is no difference in the levels of secreted IpaD, IpaB, or IpaC from the uninduced overnight cultures, meaning that the IpaD mutants continue to serve their roles in preventing uninduced protein secretion (leakage) through the T3SS injectisome.

In addition, the same *Shigella* strains described above were examined for their ability to secrete T3SS proteins under induced conditions. It is well-understood that exposure to the azo dye Congo red induces active protein secretion through the injectisome and has been suggested to mimic native activation that results from contact with human colonic epithelial cells (45). Figure 4*B* includes a representative Western blot and the resulting comparative densitometry analysis of induced protein secretion levels. As expected, a strain lacking IpaD is significantly hindered in its ability to secrete T3SS proteins following activation. Expression of IpaD^WT^, IpaD^ΔQ148^, or IpaD^ΔY149^ all complement response to Congo red and active secretion of the translocator proteins IpaB and IpaC, suggesting that the π-helix is not involved in IpaD’s role as an injectisome gatekeeper or in activation of protein secretion. The only significant difference in secretion levels is observed in IpaD between the IpaD^WT^ and the IpaD^ΔQ148^ strains with the IpaD^ΔQ148^ strain secreting less IpaD than the IpaD^WT^ strain.

**Figure 5:**
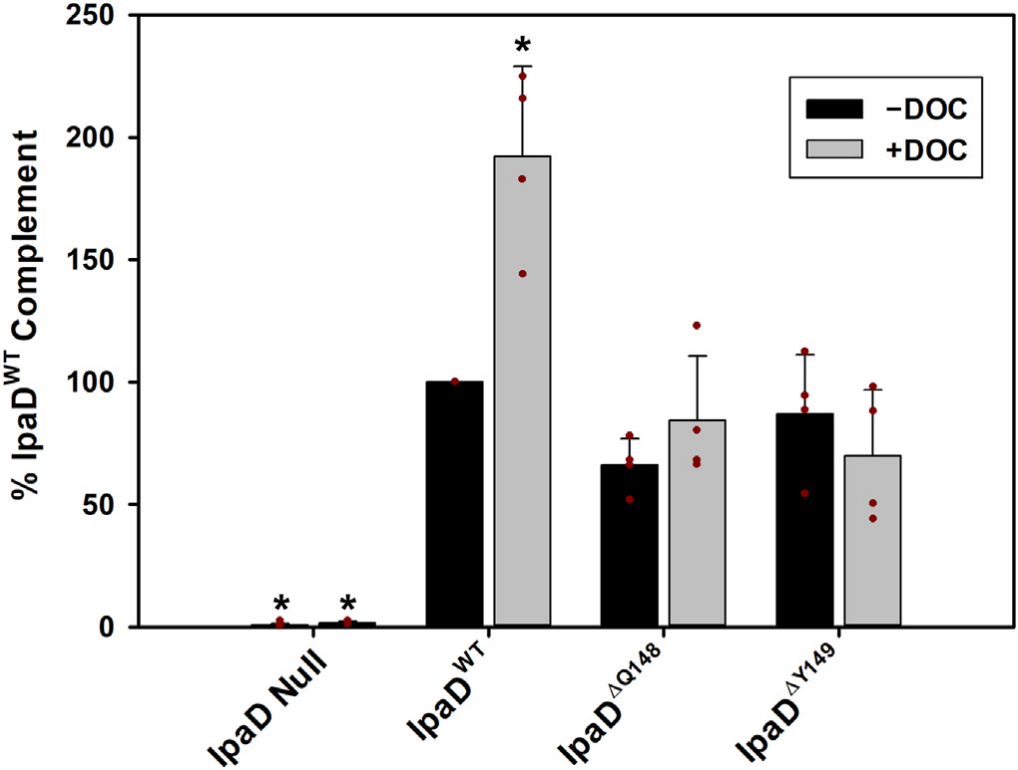
**Impact of expressing engineered IpaD π-helix deletion mutants on *Shigella* invasion phenotypes.** HeLa cell invasion phenotypes for *Shigella* strains expressing the engineered IpaD π-helix deletion mutants are presented relative to the strain expressing IpaD^WT^. As expected, *Shigella* lacking the gene for IpaD are avirulent and incubating the strain expressing IpaD^WT^ with DOC prior to exposure to the HeLa cells results in an approximately 2-fold enhancement in invasion efficiency. In contrast, while the strains expressing IpaD^ΔQ148^ and IpaD^ΔY149^ maintain WT levels of invasion in the absence of DOC, they do not demonstrate an enhancement in invasion phenotype following DOC exposure, implicating the IpaD π-helix in DOC-mediated virulence enhancement. The results are plotted as the mean ± standard deviation resulting from four independent biological replicates. ∗ indicates statistical difference from the strain expressing IpaD^WT^ in the absence of DOC (one-way ANOVA followed by a Dunnett’s post test, *p* ≤ 0.05). DOC, deoxycholate; Ipa, invasion plasmid antigen.

The cellular invasion assay has long provided one of the most sensitive and effective *in situ* means for dissecting the role(s) of *Shigella* T3SS components and their impact on virulence phenotype, including the first description of DOC-enhancement of *Shigella* virulence nearly 3 decades ago (46). We used a similar cellular invasion assay to examine the impact of removing the IpaD π-helix on *Shigella* invasion phenotype. As expected, a *Shigella* strain lacking the gene for IpaD is completely noninvasive (Fig. 5) as this strain lacks the ability to control effector/translocator protein secretion through the injectisome (Fig. 4*A*). Consistent with previous findings (34, 35), complementation with IpaD^WT^ recovers the virulence phenotype and results in a 192 ± 37% increase in invasion following exposure to DOC. More interestingly, complementation with either IpaD^ΔQ148^ or IpaD^ΔY149^ also fully recovers the strains’ invasion phenotypes, however, neither demonstrate enhanced invasion phenotype following DOC exposure (Fig. 5), supporting our hypothesis that the π-helix is essential for DOC-mediated virulence enhancement.

### High-resolution DOC-bound IpaD structures unveil the π-helix’ role in promoting productive interactions

**Figure 6:**
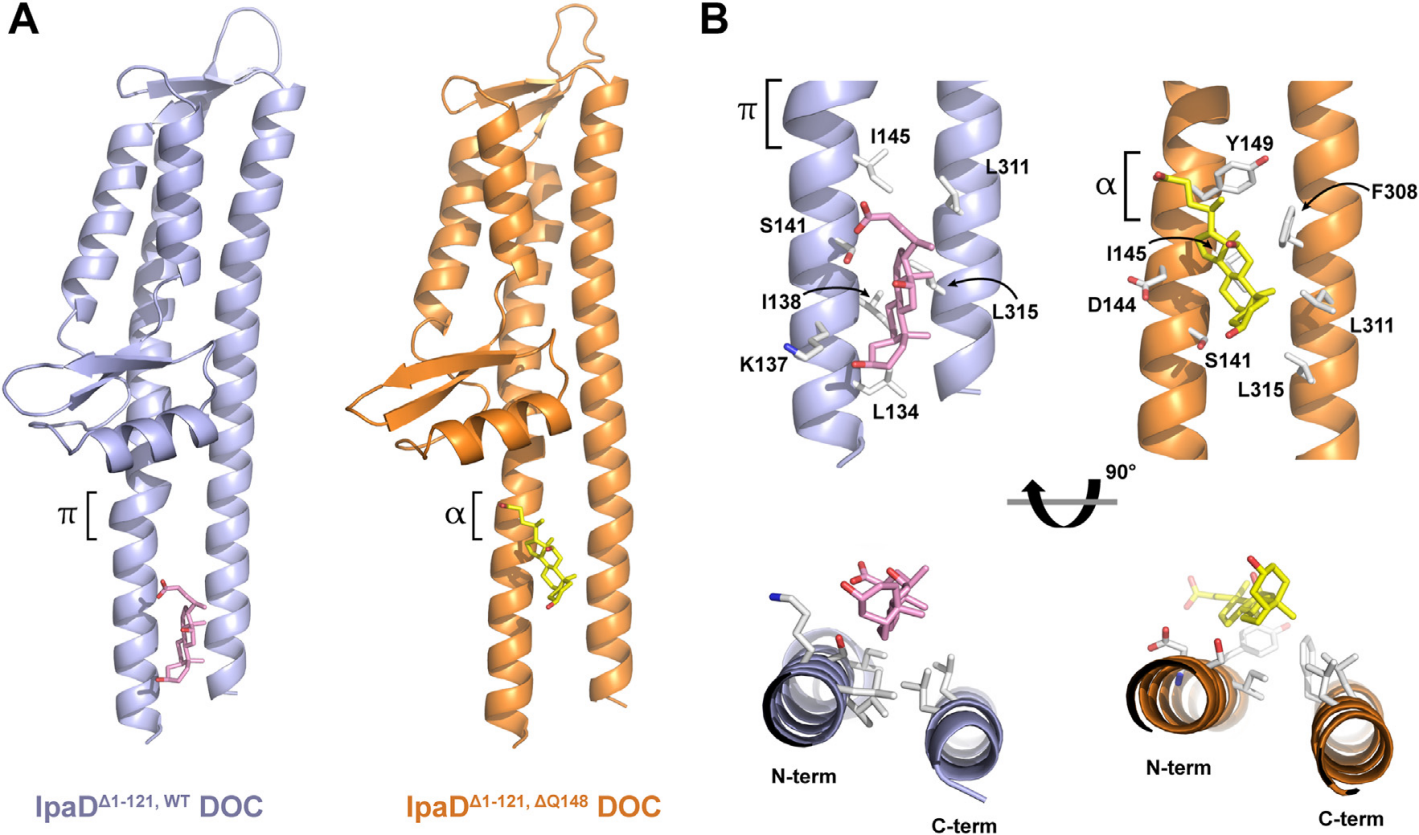
**High-resolution crystal structures uncover the role of IpaD’s π-helix in defining the DOC binding site.***A*, comparison of the IpaD^Δ1–121, WT^ (*slate*) and IpaD^Δ1–121, Q148^ (*orange*) structures shows that removal of the π-helix results in migration of the DOC binding site ∼10 Å toward the π -helix mutation. *B*, zoomed-in parallel and perpendicular views of the DOC binding sites are shown and side chains within 4.5 Å of the bound DOC ligand are included with carbon, oxygen, and nitrogen atoms colored *white*, *red*, and *blue*, respectively. DOC binding appears to be driven primarily through hydrophobic interactions in both sites as aliphatic side chains are generally oriented toward the DOC while polar and charged side chains are oriented away and exceed 4.9 Å from the nearest possible electrostatic-interacting partner in DOC. DOC, deoxycholate; Ipa, invasion plasmid antigen.

Having characterized the IpaD π-helix deletion mutants, we turned to X-ray crystallography to help uncover the mechanism(s) underlying the correlation between the π-helix in IpaD and DOC-enhanced *Shigella* virulence. Cocrystallizing the IpaD^Δ1–121, WT^ and IpaD^Δ1–121, ΔQ148^ constructs with DOC again resulted in the rapid formation of diffracting crystals that now provided DOC-bound structures of both IpaD^Δ1–121, WT^ (1.96 Å, PDB ID 8V5C) and the IpaD^Δ1–121, ΔQ148^ π-helix deletion mutant (2.20 Å PDB ID 8V5E) (Fig. 6 and Table 3). The DOC-bound IpaD^Δ1–121, WT^ structure agrees well with our previously published proteolytically truncated IpaD^WT^ DOC-bound structure (PDB ID 3R9V) with a single DOC molecule located in the hydrophobic groove formed between the two central amphipathic helices of the structure (RMSD 0.152 Å, Fig. S1) (34). A single DOC molecule is also located between the same central helices of IpaD ^Δ1–121, ΔQ148^; however, the binding location of the DOC moved distally nearly 10 Å compared to the IpaD^WT^ binding site, now interacting with the region of the protein where the π-helix once was (Figs. 6 and S2). A closer look at the side chains located within 4.5 Å of the bound DOCs shows that while some polar and charged side chains such as lysine 137 and serine 141 in the native binding site and aspartate 144 and serine 141 in the modified site of the π-helix deletion construct are in the vicinity of the DOC binding sites, they are oriented away from the bound DOC and do not appear to contribute to binding through electrostatic interactions. Instead, both DOC interactions are driven nearly exclusively through hydrophobic interactions (Fig. 6*B*). Importantly, fluorescence polarization shows that the N-terminal truncation constructs all bind DOC with equivalent affinities as those determined for the full-length proteins (Table S1), suggesting that the truncation required for crystallization does not significantly impact DOC interaction.

**Figure 7:**
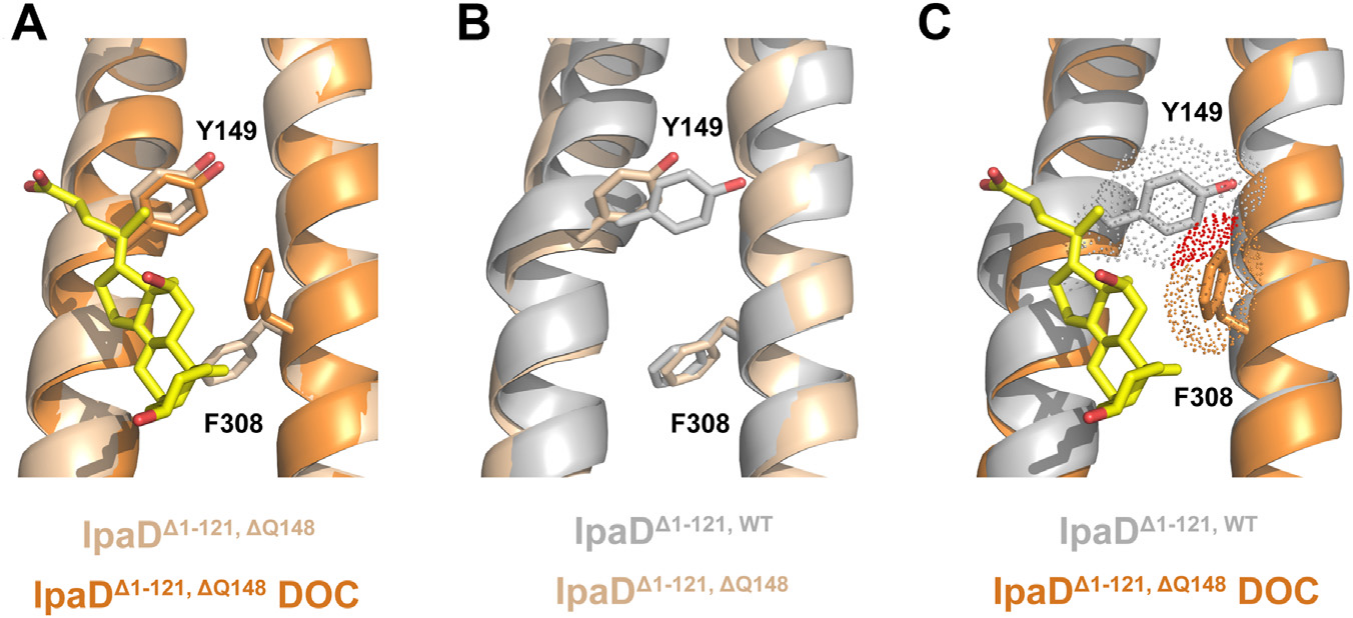
**Structural constraints provided by the IpaD π-helix prevent migration of DOC binding from the native location to the alternate site.***A*, comparing the structures of the apo (*wheat*) and the DOC bound (*orange*) IpaD^Δ1–121, ΔQ148^ mutant shows that removing the π-helix opens the alternate binding site and allows an approximately 150° rotation of the F308 side chain that then becomes an integral part of the new hydrophobic binding pocket for DOC. *B*, alignment of apo IpaD^Δ1–121, WT^ (*gray*) and IpaD^Δ1–121, ΔQ148^ (*wheat*) structures shows that removal of the π-helix tightens the associated coil in that region and pulls Y149 approximately 2.4 Å away from the helix interface. *C*, an overlay of IpaD^Δ1–121,^^WT^ (*gray*) and DOC-bound IpaD^Δ1–121,^^ΔQ148^ (*orange*) demonstrates that the π-helix prevents DOC binding in this region of IpaD^Δ1–121,^^WT^ as the π-helix backbone occludes the distal portion of the binding site and the Y149 side chain position dictated by the π-helix prevents adoption of the F308 rotamer found in the DOC-bound mutant. DOC, deoxycholate; Ipa, invasion plasmid antigen.

We first looked within the native (lower) DOC binding site for structural clues as to why it migrated with the elimination of the π-helix and found that the deletion of the π-helix results in a subtle, but noticeable, 15° rotation of the N-terminal helix away from the C-terminal helix (Fig. 6*B*). This rotation likely reduces the affinity of DOC to this site as several hydrophobic side chains previously shown to support DOC binding (34) are included in the rotation, but understanding the observed binding site migration also requires consideration of the alternate (distal) binding site that is formed with the loss of the π-helix. Superposition of the apo and DOC bound IpaD^Δ1–121, ΔQ148^ structures (Fig. 7) shows that F308 adopts an alternative rotamer conformation that forms part of the DOC binding surface when DOC is bound, permitting DOC to occupy the new site. Aligning the IpaD^Δ1–121, WT^ and IpaD^Δ1–121, ΔQ148^ structures additionally shows that Y149 sterically prevents the F308 rotamer change in the WT π-helix structure. However, removing the π-helix moves the tyrosine side chain approximately 2.4 Å away from the helix interface and the phenylalanine is no longer prevented from adopting the necessary rotamer conformation that supports DOC binding in this region. Furthermore, the π-helix backbone atoms in the WT structure also occlude the distal end of the mutant DOC binding site. Combined, these observations indicate that the alternate DOC binding site is incompatible with the IpaD^Δ1–121, WT^ structure.

### The π-helix in IpaD is essential for DOC-mediated recruitment of IpaB

**Figure 8:**
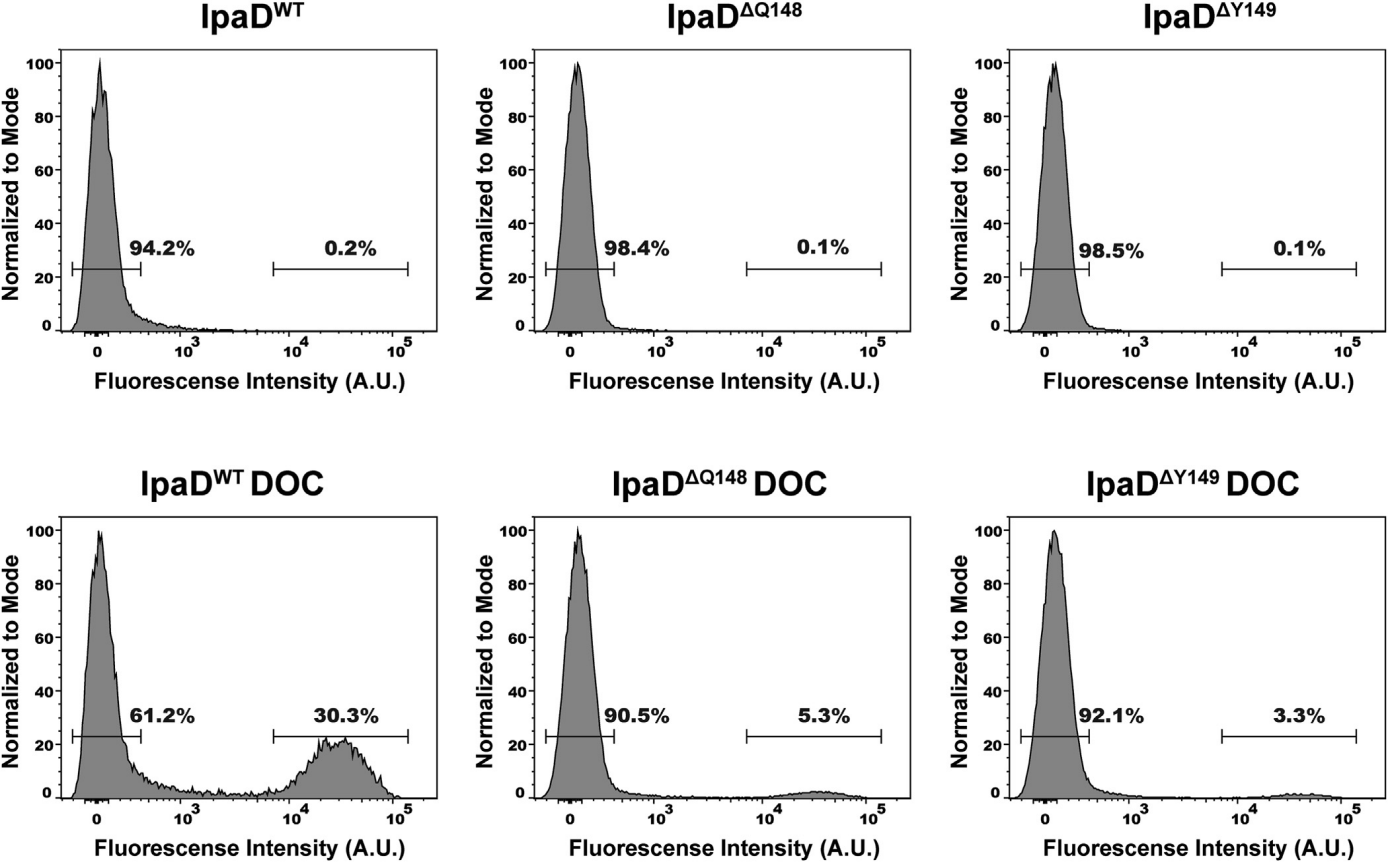
**Flow cytometry detection of surface localized IpaB.***Shigella* cultures expressing WT or the engineered π-helix deletion mutants of IpaD were chemically fixed either without prior exposure to DOC (*top row*) or following exposure to DOC (*bottom row*) and were then probed using polyclonal rabbit anti-IpaB antibodies and fluorescently conjugated anti-rabbit secondary antibodies. The fluorescence events in the histograms centered around 0 A.U. represent background fluorescence levels from individual cells and those between approximately 10^4^ and 10^5^ A.U. result from detection of IpaB that is now surface localized in response to DOC exposure. As expected, exposure of DOC to *Shigella* expressing IpaD^WT^ resulted in a robust population of the culture (30.3%) with IpaB present on the cell surface, presumably having associated with the T3SS apparatus tip in preparation of subsequent steps required for efficient host infection. Without DOC exposure, essentially none of the cells presented detectable levels of IpaB on the surface. DOC exposure of *Shigella* expressing the π-helix deletion constructs of IpaD resulted in a modest subset of the population translocating IpaB (5.3% and 3.3% for IpaD^ΔQ148^ and IpaD^ΔY149^, respectively), supporting the correlation identified between the π-helix and IpaD-mediated DOC sensitivity observed in the invasion phenotype assay. DOC, deoxycholate; Ipa, invasion plasmid antigen; T3SS, type three secretion system.

DOC exposure significantly enhances *Shigella* virulence phenotype and doing so requires the presence of the native π-helix in IpaD (Fig. 5), but the specifics surrounding the role of the π-helix in this enhancement have remained enigmatic. Based largely upon previous electron microscopy studies showing that DOC exposure recruits the translocator protein IpaB to the tip of the injectisome (28), we hypothesized that the π-helix ensures “proper” DOC binding to IpaD and that this results in recruiting IpaB to the T3SS needle tip as the first step in the injectisome maturation process. The *Shigella* strains lacking IpaD, complemented with IpaD^WT^, and those complemented with the IpaD^ΔQ148^ and IpaD^ΔY149^ mutants were assessed for surface localized IpaB using rabbit generated primary antibodies and fluorescently conjugated secondary antibodies that were detected using flow cytometry (Fig. 8). As expected, the IpaD^WT^, IpaD^ΔQ148^, and IpaD^ΔY149^ strains all lacked IpaB at the cell surface prior to DOC exposure (0.2%, 0.1%, and 0.1% of the population, respectively). Following DOC exposure, however, 30.3% of the IpaD^WT^ population was detected with IpaB on the cell surface. Exposing the IpaD^ΔQ148^, and IpaD^ΔY149^ strains to DOC resulted in only 5.3% and 3.3% of the population with IpaB detected on the cell surface, respectively, demonstrating the importance of the IpaD π-helix in responding to DOC exposure. Control conditions including an IpaB null *Shigella* strain and probing of the IpaD^WT^ strain with primary or secondary only antibody solutions prior to and following DOC exposure confirm specific detection of surface localized IpaB in these experiments (Fig. S3).

## Discussion

*Shigella* relies entirely on their T3SS for virulence and specifically call for its action during the early stages of infection to invade and colonize its human host (6, 7, 8). Transcriptional regulation, environmental sensing, T3SS apparatus maturation, and protein secretion control are all key elements in a carefully orchestrated series of events that ensures *Shigella* are optimally prepared for contact with the hosts’ colonic epithelial cells while preventing wasteful secretion of its limited supply of effector proteins during transit. The findings presented here describe the impact of the secondary bile salt DOC on regulation/maturation of the *Shigella* T3SS and elucidate the molecular basis for DOC recognition by IpaD that results in enhanced *Shigella* virulence. More specifically, this study expands upon our previous findings identifying a π-helix in IpaD (34, 35) and shows that it is a critical element in DOC-mediated *Shigella* virulence enhancement. π-helices such as this one were once thought to be exceptionally rare, but have since been found in approximately 15% of known protein structures and are believed to evolve from a residue insertion within an α-helix and remain despite their thermodynamic disadvantage as they are generally associated with critical functions (36, 37, 38).

Studying structures of IpaD^WT^ suggests that glutamine 148 and tyrosine 149 may each serve as the hypothesized insertion residues responsible for the π-helix formation and deletion mutations were generated for each to attempt to revert the π-helix to an α-helix and provide constructs necessary to determine the specific role(s) of the π-helix in IpaD/*Shigella*. Biophysical characterization of both IpaD^ΔQ148^ and IpaD^ΔY149^ shows that the mutants maintain essentially WT global secondary structure composition (Fig. 1 and Table 1) and that they continue to bind DOC with similar (low μM) affinity as IpaD^WT^ (Fig. 2), suggesting that either the deletion mutations failed to remove the π-helix or that its removal is not detrimental to either of these factors. Fortunately, a 3.00 Å structure of IpaD^ΔQ148^ verified the removal of the π-helix (conversion to α-helix) and minimal impact on the global structure of the mutant (Fig. 3), consistent with the CD data for both constructs (Fig. 1).

Next, *in vivo* impact of the IpaD π-helix was assessed in *Shigella* strains expressing the engineered mutants. Interestingly, the *Shigella flexneri* IpaD^ΔQ148^ and IpaD^ΔY149^ strains both maintain their ability to prevent unwanted protein “leakage” through the T3SS apparatus (Fig. 4*A*), demonstrating that IpaD’s role in preventing premature secretion is not impacted by deletion of the π-helix. This observation is important as it begins to delineate IpaD functions which are and are not impacted by the proposed evolutionary appearance of its π-helix, with secretion control responsibilities likely predating its DOC recognition pathway. In the context of an infection, this IpaD-regulated presecretion state represents bacteria that have been ingested and temperature-induced transcription of the corresponding T3SS genes supports the assembly of the injectisome capped by IpaD (20, 21, 47). We propose that once *Shigella* enters the high concentration of bile salts in the small intestine, DOC binds to IpaD and this interaction drives the observed DOC-mediated virulence enhancement that prepares them for infection of the downstream colonic epithelium. Figure 5 shows that this is the point in *Shigella* pathogenesis where the π-helix becomes critical as both of the *Shigella* strains expressing the engineered mutants lose DOC impact on invasion phenotype. Together, these findings provide the first direct correlation between the IpaD π-helix, DOC, and invasion phenotype, leaving the important question as to what specific role the π-helix plays in this pathway. We have long suggested that the interaction of DOC with IpaD results in the first of a series of maturation steps at the T3SS apparatus needle tip by recruiting the hydrophobic translocator IpaB and poising the apparatus for interaction with the host cell membrane (28, 29, 33, 48). Flow cytometry detecting translocated IpaB confirms that exposing *Shigella* cells to DOC does in fact recruit IpaB, but more importantly, *Shigella* expressing the IpaD π-helix deletion mutants failed to efficiently recruit IpaB to the cell surface in response to DOC exposure (Fig. 8). More specifically, these findings delineate that while the π-helix is not required for controlling IpaB (or other translocator) secretion through the apparatus (Fig. 4), it is essential for translating DOC exposure to its stable recruitment (Fig. 8) and virulence enhancement (Fig. 5). This priming presumably helps guide *Shigella* to infect the downstream colon while contributing to the impressive efficiency with which *Shigella* infection occurs, but the mechanism was not clear and seems to contradict the DOC binding data showing that deletion of the IpaD π-helix does not impact affinity for DOC. X-ray crystal structures of DOC-bound IpaD^WT^ and DOC-bound IpaD^ΔQ148^ were critical for resolving this apparent conflict by providing the first molecular insight into the role of IpaD’s π-helix in the DOC-mediated *Shigella* virulence enhancement pathway. More specifically, comparing these structures shows that eliminating the π-helix does not eliminate DOC binding, but instead causes the single binding site for DOC to migrate approximately 10 Å, now docking in the cleft between the two central helices where the elimination of the π-helix occurred (Figs. 6 and 7).

This finding was unexpected and particularly exciting as the role of the π-helix has long been hypothesized to be a hinge point for conformational changes driven by DOC interaction (33, 34, 35), not understood to play a direct role in determination of the DOC binding site. Notably, while very modest conformational changes are observed in the crystal structures as a result of DOC binding to either the IpaD^Δ1–121, WT^ or IpaD^Δ1–121, ΔQ148^ structures, the removal of the π-helix in both the apo and bound conditions results in a lateral shift of the central helices relative to the WT structure, resulting in a 7 Å translation of the distal ends of the structures (Fig. S4). While the specific implications of these conformational impacts are not entirely clear, it does suggest that the π-helix may indeed provide a degree of conformational flexibility that is sensitive to appropriate DOC binding but is not fully captured in the crystal structures. A full understanding of the structural impact of the π-helix and DOC binding may require *in vivo* visualization in the context of the pentameric IpaD tip complex (42). While *in vivo* assessment of the tip complex structure is ambitious, advancements in cryo-electron tomography data collection and image processing have made it feasible and in fact, Guo *et al.* recently solved a 3.90 Å structure of the *Salmonella* T3SS needle tip complex comprised of a homopentamer of the IpaD homolog, SipD (49). Bile salt influence was not considered in this study, however, because despite the fact that SipD is the only T3SS tip protein in addition to IpaD to contain a π-helix (50, 51) *Salmonella* does not respond to bile salt exposure with virulence enhancement or apparatus maturation. In fact, DOC exposure decreases *Salmonella* virulence *via* transcriptional downregulation of key T3SS genes (52). Nevertheless, this seminal tip complex structure does provide important opportunities for comparison among T3SSs and highlights the potential to observe IpaD in a context that most certainly influences the impact of DOC interaction.

Ultimately, the findings presented in this study highlight the pivotal role of the IpaD π-helix in interaction with DOC, IpaB recruitment, and subsequent enhancement of virulence phenotype in *Shigella*. The unexpected discovery of the π-helix role in occluding an alternate, nonproductive, binding site for DOC reveals the complexity of the DOC initiated response pathway and the evolutionary divergence of environmental influence on T3SS activity. Together, these novel findings advance our understanding of T3SS function and *Shigella* pathogenesis and guide studies to further elucidate details of this complex pathway while identifying a high-priority target for the development of nonantibiotic agents to combat the rapidly growing number of antibiotic-resistant *Shigella* strains.

## Experimental procedures

### Materials

WT *S. flexneri* corresponds to the serotype 2a 2457T strain originally isolated in 1954 (53). FITC-DOC and rabbit polyclonal antibodies against IpaB, IpaC, and IpaD were generous gifts from William and Wendy Picking (University of Missouri). The anti-IpaB, IpaC, and IpaD antibodies were all provided as antisera and diluted 1:500 prior to use in the described experiments. Selectivity and sensitivity of each antibody was evaluated by performing SDS-PAGE followed by Western blot analyses against 80 ng of the appropriate recombinant, purified target protein, as well as against a total protein extract obtained from *Shigella*. IpaD and IpaB null *Shigella* strains were generated by Philippe Sansonetti (Institut Pasteur, Paris, France) *via* nonpolar kanamycin resistance cassette transposon insertion into *S. flexneri* M90t. *E. coli* strains, the pET15b expression plasmid, and 2× ligation mix were from Novagen. Restriction enzymes, the pTYB21 expression plasmid, chitin resin, PCR buffer, and Phusion High-Fidelity polymerase were purchased from New England Biolabs. Oligonucleotide primers were from Integrated DNA Technologies. The Superdex size-exclusion and Q FF columns were purchased from General Electric. Defibrinated sheep red blood cells were from Colorado Serum Company. All other solutions and chemicals were of reagent grade.

### Cloning

The gene encoding IpaD was amplified from the isolated virulence plasmid of *S. flexneri* 2457T using primers with appropriate restriction sites to incorporate the gene into pET15b and pWPsf4 expression plasmids. The *ipaD*^*ΔQ148*^ and *ipaD*^*ΔY149*^ mutants were generated from the resulting constructs using 5′ phosphorylated primers to eliminate the codon targeted for deletion *via* inverse PCR. The N-terminal truncation constructs of WT and the π-helix deletion mutants (ΔQ148 and ΔY149) of IpaD used for protein crystallization were also generated using inverse PCR and were cloned into pTYB21 for expression. Each of the newly engineered constructs was verified by Sanger sequencing prior to transformation into *E. coli* or IpaD null *S. flexneri* strains.

### Protein expression and purification

Each of the *ipaD* pET15b and *ipaD* pTYB21 constructs was transformed into *E. coli* Tuner (DE3) cells for expression. Each *E. coli* strain was grown overnight in LB broth containing 0.1 mg/ml ampicillin to maintain the expression plasmid. The overnight cultures were used to inoculate larger growths in terrific broth which were grown at 37 °C and 200 rpm to an absorbance (*A*_600_) of 0.8 prior to cooling to 17 °C and induction with 1 mM IPTG. The induced cultures were maintained at 17 °C and 200 RPM for 20 h prior to isolation by centrifugation. The IpaD proteins expressed from pET15b were resuspended in Ni^2+^ purification binding buffer (20 mM Tris, 500 mM NaCl, and 5 mM imidazole, pH 7.9) and lysed using a probe sonicator. The soluble proteins were separated from the insoluble cell components *via* centrifugation and the 6× His-tagged IpaD constructs were initially purified using nickel chelation chromatography. The purified IpaD protein eluted from the nickel column was then dialyzed into 10 mM Tris 10 mM NaCl pH 7.9 and further purified using a GE Q FF anion exchange column and AKTA purifier. Finally, the anion exchange elution fractions containing IpaD were concentrated using a Sartorious Vivaspin Turbo 15 concentrator with a 30 kDa molecular weight cutoff and run over a Superdex 200 size exclusion column into PBS, pH 7.4. Following expression, cells expressing chitin binding domain—IpaD^Δ1–121^ constructs expressed from pTYb21 were pelleted by centrifugation, resuspended in 20 mM Tris, 500 mM NaCl, pH 7.9, and lysed using a probe sonicator. The soluble protein was collected by centrifuging the post sonicated cellular lysate prior to exposure to a chitin column and purification *via* release of the IpaD constructs by DTT mediated on-column cleavage of the intein domain. The elution fractions containing IpaD were dialyzed against 10 mM Tris, 10 mM NaCl, pH 7.9 to allow interaction with a Q Sepharose FF anion exchange column and elution with a NaCl gradient. The purified IpaD fractions were concentrated using a Sartorious Vivaspin Turbo 15 concentrator with a 30 kDa molecular mass cutoff for final purification using a Superdex 200 16/600 size exclusion column equilibrated with ddH_2_O.

### Crystallization

Crystallization of IpaD^Δ1–121^^, WT^ and IpaD^Δ1–121,^ ^ΔQ148^ was performed using standard vapor diffusion methods and protein concentrations of approximately 10 mg/ml in ddH_2_O and 1.33:1, 1:1, and 1:1.33 protein:well solution drop ratios incubated at room temperature (21–22 °C). Crystals grew within 1 week in 0.1 M Hepes pH 7.5, 18 to 20 mM magnesium chloride hexahydrate, 20% w/v Poly(acrylic acid sodium salt) 5100. Cocrystallization with DOC was achieved by adding 0.3 mM DOC acid sodium salt into the crystallization well solution. Harvested crystals were soaked in a 7.5% glycerol cryo-solution and flash frozen in liquid nitrogen in preparation for shipment to the Stanford Synchrotron Radiation Lightsource for remote data collection.

### X-ray diffraction data collection and structure determination

Crystallographic data were collected to 2.70 Å (IpaD^Δ1–121, WT^), 3.00 Å (IpaD^Δ1–121, ΔQ148^), 1.96 Å (IpaD^Δ1–121, WT, DOC^), and 2.20 Å (IpaD^Δ1–121, ΔQ148, DOC^) at the Stanford Synchrotron Radiation Lightsource beamline 9-2. The diffraction data were processed using HKL3000. The crystals of IpaD^Δ1–121, WT^, IpaD^Δ1–121, ΔQ148^, and IpaD^Δ1–121, WT, DOC^ belong to the P2_1_ space group and IpaD^Δ1–121, ΔQ148, DOC^ crystals belong to the P 2_1_2_1_2_1_ space group. The IpaD^Δ1–121, WT^ structure was solved using molecular replacement with the previously published truncated IpaD structure (PDB code 2J0N) as a search model (40). All other IpaD structures solved in this study utilized molecular replacement with the IpaD^Δ1–121, WT^ structure as a search model. Coot (https://www2.mrc-lmb.cam.ac.uk/personal/pemsley/coot/) was used for model building (54). The PHENIX software (https://phenix-online.org/) package was used for molecular replacement, refinement, and map calculations (55). All structure figures were rendered using PyMOL (The PyMOL Molecular Graphics System, version 3.0 Schrödinger, LLC, https://www.pymol.org/).

### Far-UV CD

Far-UV CD spectra and secondary structure thermal stability profiles were collected for IpaD^WT^ and each of the engineered IpaD π-helix deletion mutants in the absence and presence of 1 mM DOC. All data were collected using a JASCO model J-1500 spectropolarimeter equipped with a temperature-controlled sample chamber. CD spectra were collected from 190 nm to 260 nm at 10 °C using 0.1 cm quartz cuvettes, 0.1 nm data sampling, a 50 nm/min scan rate, and a 1 s data integration time. Secondary structure thermal stability profiles were collected in the same 0.1 cm quartz cuvettes by monitoring the CD signal at the 222 nm spectra minimum while the solution temperature was increased from 10 to 90 °C at a rate of 0.3 °C/min. Measurements were performed on 0.5 mg/ml protein solutions in PBS, pH 7.4. CD signals were normalized by converting to mean residue molar ellipticity and were analyzed using the Dichroweb software (http://dichroweb.cryst.bbk.ac.uk) package with the CDSSTR analysis method and reference set 7 (56, 57). Thermal unfolding transition temperatures were determined by fitting the profiles to a 5-parameter sigmoidal function to determine the transition inflection point.

### IpaD FITC-DOC fluorescence polarization

The FITC-DOC binding assay was performed with minor modifications to the previously described protocol (27, 35). Specifically, 25 nM FITC-DOC was incubated with purified IpaD (0–30 mM) at 25 °C for 1 h in PBS pH 7.4. Each condition was transferred to 3 wells of an opaque flat-bottom 96-well plate and the fluorescence polarization of the FITC-DOC was measured using a Synergy H4 fluorescence plate reader. The plate reader is equipped with polarizers and bandpass filters providing 485 ± 10 nm excitation and detection of fluorescence emission at 528 ± 10 nm. The change in polarization in response to IpaD concentration was plotted and fit to a single site saturation binding model in SigmaPlot 12 (https://grafiti.com/sigmaplot-detail/). The apparent dissociation constants (K_d_) were determined from the fitted curves.

### Uninduced secretion of *Shigella* translocators

The uninduced secretion (leakage) assay was performed as described previously (35, 39, 43). Briefly, the *S. flexneri* strains expressing IpaD^WT^ or the IpaD π-helix deletion mutants were grown overnight in tryptic soy broth (TSB) containing 0.1 mg/ml ampicillin and 0.05 mg/ml kanamycin. The bacteria were separated from the culture media *via* centrifugation and the bacterial pellet was suspended 1× SDS sample buffer, boiled, and separated using SDS PAGE. The secreted proteins in the culture supernatant were analyzed by precipitation with 10% trichloroacetic acid, rinsed with acetone, resuspended in SDS sample buffer, and separated using SDS-PAGE. The proteins were then transferred from the SDS-PAGE gels to polyvinylidene difluoride membranes for analysis *via* Western blot. The relative levels of secreted T3SS proteins were determined using rabbit primary antibodies against IpaD, IpaB, and IpaC followed by exposure to Alexa 647-conjugated goat anti-rabbit secondary antibodies. A commercial Alexa488-conjugated mouse monoclonal antibody against the cytoplasmic enzyme GAPDH was used as a control to ensure that the proteins observed in the supernatant were secreted and not the result of cell lysis. Fluorescence intensities were measured using BioRad ChemiDoc fluorescence imager, and the protein bands were quantified *via* fluorescence densitometry.

### Induced secretion of *Shigella* translocators

The Congo red induced secretion assay was performed as described previously (43, 58, 59). Briefly, *Shigella* strains were streaked onto tryptic soy agar (TSA) plates containing 0.025% Congo red and incubated overnight at 37 °C. Subsequently, 10 ml TSB cultures were started from the TSA-Congo red plates and were grown to and *A*_600_ of 1.0 in TSB containing 0.1 mg/ml ampicillin and 0.05 mg/ml kanamycin to maintain the expression and virulence plasmids, respectively. The cultures were then cooled and the bacteria collected *via* centrifugation at room temperature (21–22 °C) prior to resuspension in room temperature (21–22 °C) sodium phosphate buffer (10 mM NaH_2_PO_4_, 150 mM NaCl, pH 7.2). Congo red was added to a final concentration of 0.3 mg/ml and the bacterial solutions were incubated at 37 °C for 30 min to induce T3SS protein secretion. The bacteria were pelleted *via* centrifugation, and the supernatant was collected and analyzed for IpaB, IpaC, and IpaD by Western blot as described in detail for the uninduced protein secretion assay.

### Bacterial invasion of epithelial cells

The invasion assay was performed as described previously (60, 61) with minor modifications to support evaluation of the effect of DOC. Briefly, sterile 24-well plates were seeded with passaged HeLa cells and incubated overnight in Dulbecco’s modified Eagle’s medium (DMEM) supplemented with 10% fetal calf serum, penicillin, and streptomycin at 100% relative humidity, 37 °C, and 5% CO_2_. The *S. flexneri* strains were streaked onto TSA plates containing 0.025% Congo red and grown overnight at 37 °C. A small number of isolated colonies from each plate were used to inoculate 10 ml of TSB containing appropriate antibiotics to maintain the transformed plasmid. The cultures were grown to an *A*_600_ of approximately 0.25 at 37 °C and 200 RPM. The cultures were split with half of the culture transferred to a sterile flask containing DOC to a final concentration of 1.0 mg sodium DOC/ml of culture. The other half of the culture was moved to an identical sterile flask lacking DOC. The cultures continued to grow at 37 °C for 30 min prior to introduction of identical bacterial loads (multiplicity of infection = 10) to HeLa cells recently rinsed with antibiotic free 0.45% glucose DMEM solution. The plates were centrifuged at 188*g* to initiate contact between the bacteria and HeLa cells and then incubated for 30 min at 100% relative humidity, 37 °C, and 5% CO_2_ to allow cellular invasion. Each well was then treated with DMEM solution containing 50 μg/ml gentamicin to selectively kill extracellular bacteria. The number of bacteria that had invaded the HeLa cells was determined by lysing them with 1% agarose in water and then overlaying a 2× LB agar solution. The resulting colonies were enumerated after incubation overnight at 37 °C and compared to determine the relative invasion of the *S. flexneri* strains both in the presence and absence of DOC.

### Flow cytometry detection of surface localized IpaB

TSA-Congo red plates were streaked with *S. flexneri* strains expressing IpaD^WT^, IpaD^ΔQ148^, or IpaD^ΔY149^. Isolated colonies were used to inoculate TSB media containing the appropriate antibiotics and grown to an *A*_600_ of approximately 0.25 at 37 °C and 200 RPM. The culture was then split into two smaller growth flasks, one containing DOC to a final concentration of 1 mg/ml and the other lacking DOC. The cultures were grown for an additional 30 min and were harvested by centrifugation at 3738*g* and 4 °C. The cell pellets were resuspended in PBS containing 5% Tween 20 and centrifuged again prior to chemical fixation by resuspending in PBS containing 4% formaldehyde followed by a 15-min incubation at room temperature (21–22 °C). The solution was quenched using 2.5 M Tris pH 7.4 and rinsed three times with PBS containing 5% Tween 20 prior to a final rinse with PBS. The cells were again centrifuged to separate the bacteria and the pellet resuspended/incubated in Pierce Protein-Free PBS blocking buffer prior to treatment with rabbit polyclonal anti-IpaB antibodies in Tween 20-blocking buffer (10% Piece protein-free blocking buffer and 0.05% Tween 20 in PBS). The treated cells were then rinsed with blocking buffer containing Tween 20, incubated with Alexa Fluor 647-conjugated goat anti-rabbit secondary antibodies, rinsed with blocking buffer containing Tween 20, and finally rinsed with PBS. Control conditions include *Shigella* cultures treated identically to the description above, but lacking exposure to either the primary or secondary antibodies as well as a *S. flexneri* strain that does not express IpaB. Each of the engineered cell lines (±DOC) were then analyzed using a FACSAria cell sorter equipped with a 640 nm laser excitation source and 660/20 bandpass emission filter for detection of Alexa Fluor 647-labeled IpaB. The resulting cytometry data were gated identically and evaluated using the FlowJo software (https://www.flowjo.com) package.

## Data availability

Main text data are contained within this article and structure coordinates are deposited in the RCSB Protein Data Bank (PDB IDs: 8V7S, 8V7Q, 8V5C, and 8V5E). Supplemental data are in the corresponding Supporting Information document. Correspondence and requests for materials should be addressed to the corresponding author (nick.dickenson@usu.edu).

## Supporting information

This article contains supporting information.

## Conflict of interest

The authors declare that they have no conflicts of interest with the contents of this article.
