# Supplementary material for: Structural and functional characterization of the IpaD π-helix reveals critical roles in DOC interaction, T3SS apparatus maturation, and Shigella virulence
Source: J Biol Chem. 2024 Jul 28;300(9):107613. doi: 10.1016/j.jbc.2024.107613 (PMC11400957; doi:10.1016/j.jbc.2024.107613)
Supplement: Supplemental Figures S1–S4 and Table S1 [file mmc1.pdf]

Structural and functional characterization of the IpaD π-helix reveals roles in DOC interaction, T3SS apparatus maturation, and *Shigella* virulence

Samuel A. Barker[1], Abram R. Bernard[1], Yalemi Morales[1], Sean J. Johnson[1], Nicholas E. Dickenson[1,*]

From the [1]Department of Chemistry and Biochemistry, Utah State University, Logan, Utah

* For correspondence: Nicholas E. Dickenson, nick.dickenson@usu.edu

Supporting Information contents

Figure S1. Alignments of previously published IpaD structures and those solved in this study.

Figure S2. IpaD/DOC omit map.

Figure S3. Flow cytometry control experiments.

Figure S4. Global structure analysis of IpaD π-helix mutant structures.

Table S1. Apparent binding affinities between FITC-DOC and N-terminally truncated IpaD mutants

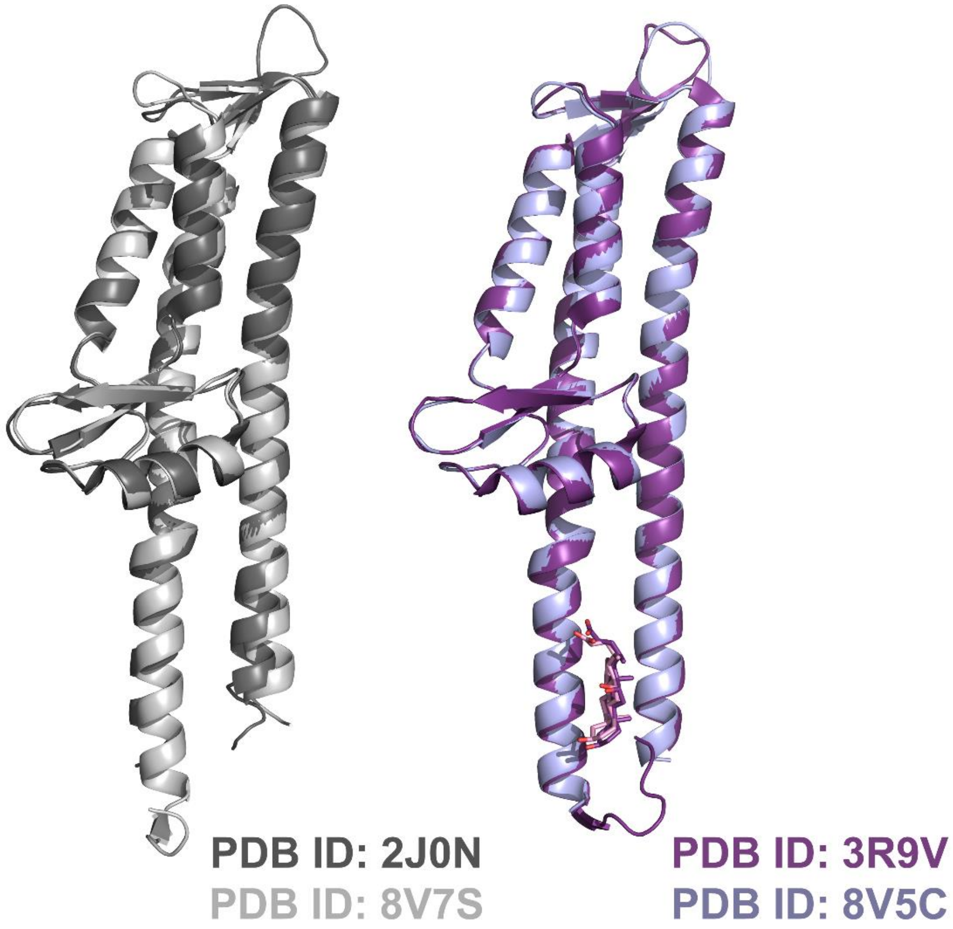

Figure S1. Left: Global Cα alignment of the previously published crystal structure of a proteolytically cleaved IpaD (2J0N, dark gray) and the IpaD$^{\Delta 1\text{-}121,\ WT}$ crystal structure solved in this study (8V7S, light gray). RMSD = 0.485 Å. Right: Global Cα alignment of the previously published crystal structure of proteolytically cleaved IpaD bound to DOC (3R9V, purple) and the DOC-bound IpaD$^{\Delta 1\text{-}121,\ WT}$ crystal structure solved in this study (8V5C, slate). RMSD = 0.152 Å.

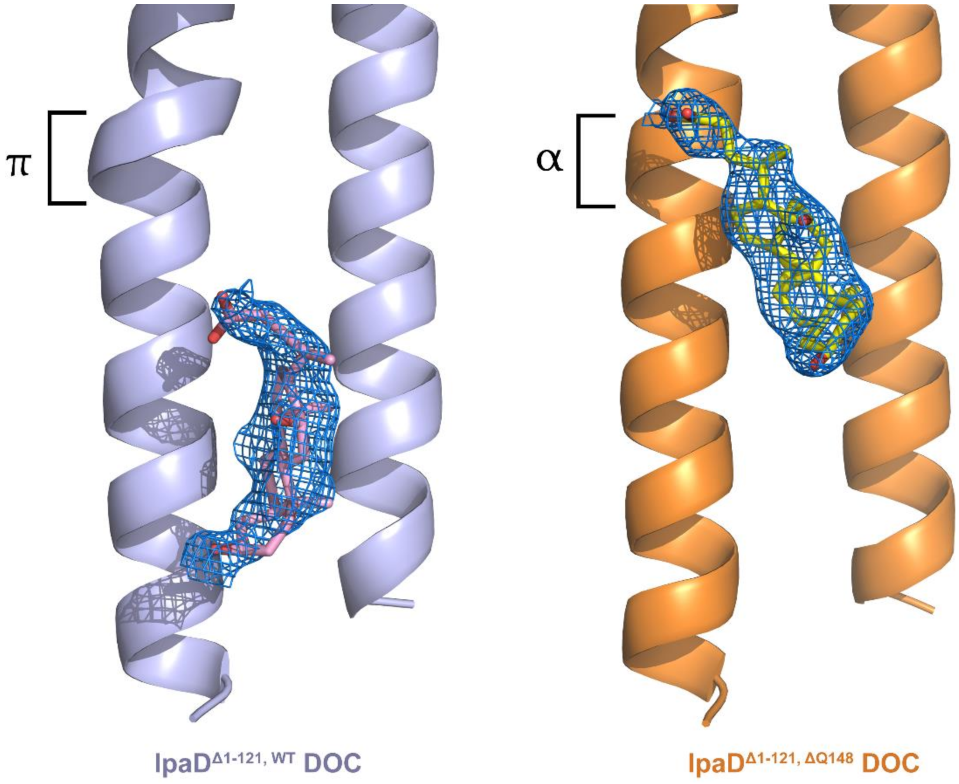

Figure S2. Simulated annealing omit maps of DOC bound to IpaD$^{Δ1\text{-}121,\ WT}$ (slate) and IpaD$^{Δ1\text{-}121,\ ΔQ148}$ (orange), contoured at 1σ.

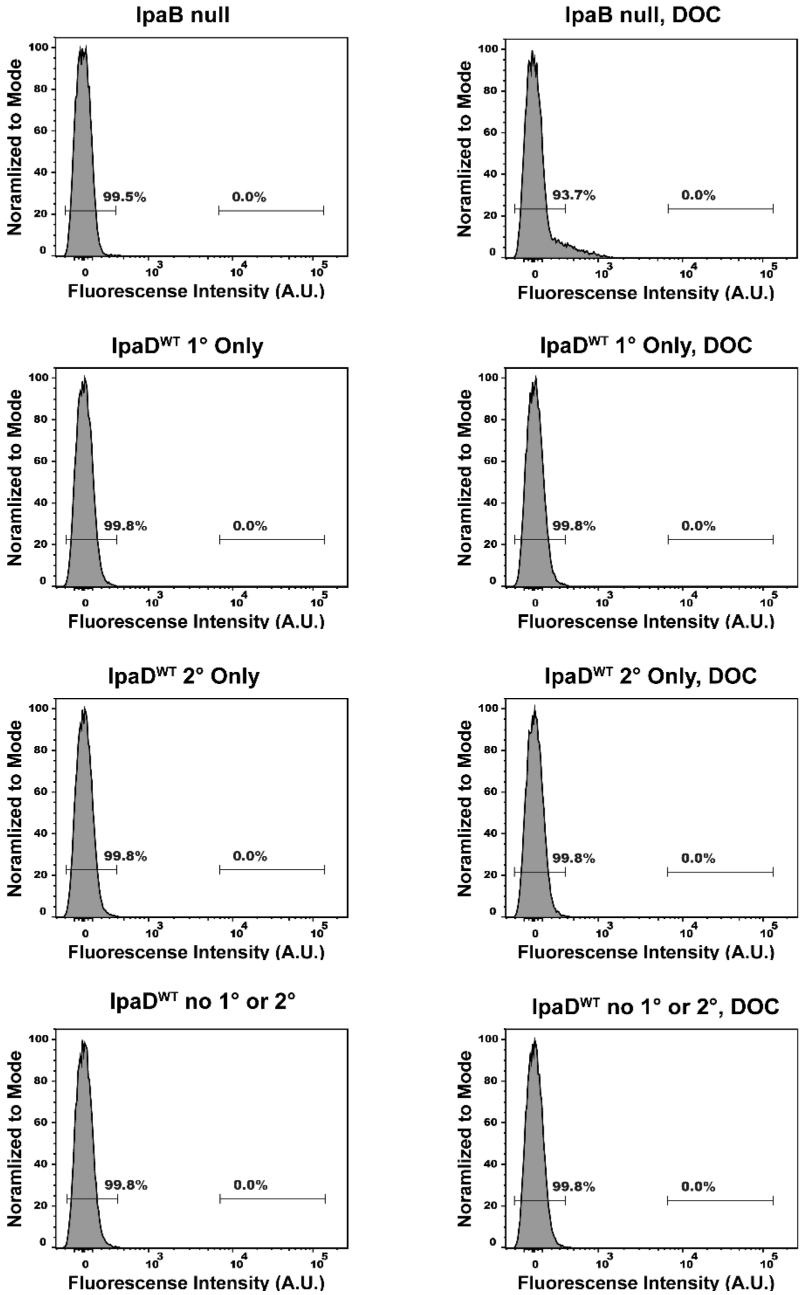

Figure S3. Control experiments ensuring specificity of IpaB detection by flow cytometry. An *S. flexneri* strain lacking the gene for IpaB (IpaB null) and a strain natively expressing IpaB and expressing IpaD^{WT} from the pWPsf4 expression plasmid were chemically fixed either without prior exposure to DOC (Left Column) or following exposure to DOC (Right Column). As expected, no signal associated with IpaB on the cell surface was detected in the control conditions for cells lacking IpaB expression (IpaB null), treated with only the primary rabbit antibodies against IpaB (1° only), only treated with fluorescently conjugated goat anti-rabbit secondary antibodies (2° only), or not exposed to any antibodies prior to assessment (no 1° or 2°). The fluorescence events in the histograms centered around 0 A.U. represent background auto-fluorescence levels with positive detection of IpaB expected between approximately 10^4 and 10^5 A.U, as seen in Figure 8 of the main text.

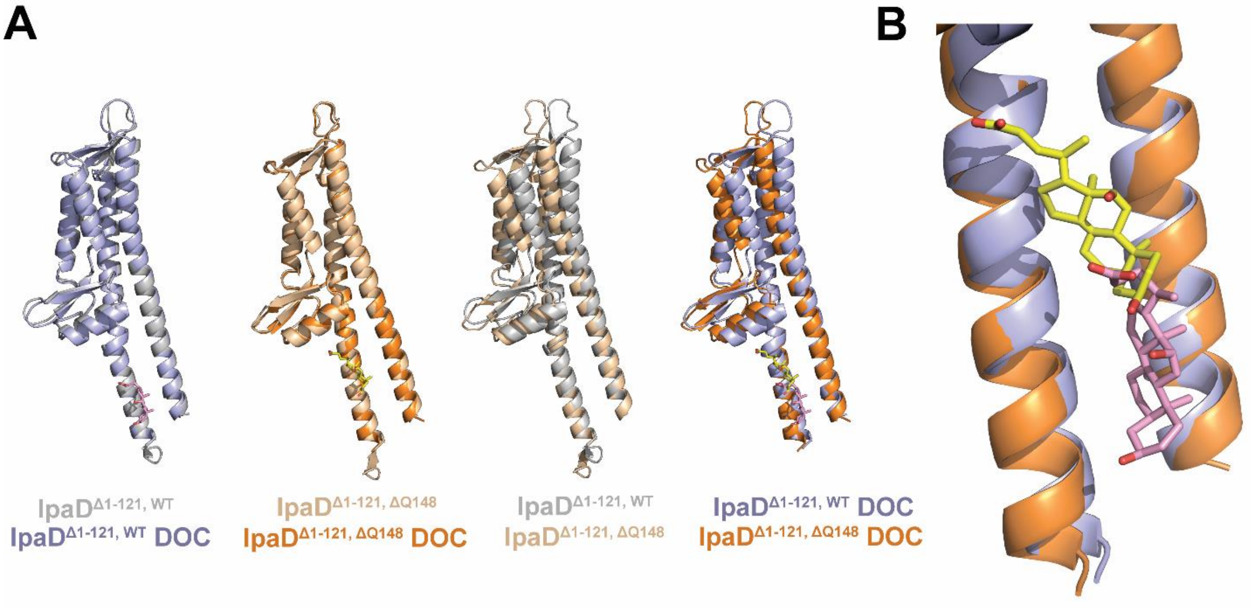

Figure S4. Alignments of the apo and DOC-bound IpaD^{WT} and IpaD$^{\Delta 1\text{-}121,\ \Delta Q148}$ mutant structures solved in this study. **(A)** DOC binding results in minimal global conformational impact of either IpaD$^{\Delta 1\text{-}121,\ WT}$ or IpaD$^{\Delta 1\text{-}121,\ \Delta Q148}$ (left two alignments) while elimination of the π-helix in the IpaD$^{\Delta 1\text{-}121,\ \Delta Q148}$ mutant results in a nearly 7Å lateral translation of the central helices originating at the location of the π-helix deletion (right two alignments). **(B)** A close-up view of the DOC binding location in IpaD$^{\Delta 1\text{-}121,\ WT}$ (slate, pink) and IpaD$^{\Delta 1\text{-}121,\ \Delta Q148}$ (orange, yellow), demonstrating that the DOC binding sites overlap and are mutually exclusive.

Table S1. Fluorescence polarization-derived binding affinities between N-terminally truncated IpaD π-helix mutants and deoxycholate.

IpaD Construct	K_d (μM ± SD)[a]
$IpaD^{\Delta 1\text{-}121,\ WT}$	6.2 ± 1.1
$IpaD^{\Delta 1\text{-}121,\ \Delta Q148}$	5.0 ± 1.8
$IpaD^{\Delta 1\text{-}121,\ \Delta Y149}$	5.5 ± 1.2

[a]Apparent K_d values between FITC-DOC and the N-terminally truncated IpaD constructs used in this study. Values are reported as the mean ± standard deviation of the apparent K_ds calculated from three independent biological replicates. No difference is observed in DOC affinity among these N-terminally truncated IpaD constructs when compared to the full-length constructs in Table 1 of the main text. (one-way ANOVA, $p \leq 0.05$).
